# Supplementary material for: Nociceptor neurons control pollution-mediated neutrophilic asthma
Source: eLife. 2026 Mar 27;13:RP101988. doi: 10.7554/eLife.101988 (PMC13030891; doi:10.7554/eLife.101988)
Supplement: MDAR checklist [file elife-101988-mdarchecklist1.docx]

**Materials Design Analysis Reporting (MDAR) Checklist for eLife**

**Materials**

- **Newly created materials:** Not applicable. No new materials (e.g., new reagents, organisms, or chemical compounds) were generated.
- **Antibodies:** Antibodies used for flow cytometry are reported in the Methods with **clone IDs,** RRIDs and **suppliers** (BioLegend or Thermo Fisher Scientific), with **catalog numbers provided where stated** (e.g., anti-Ly6G Alexa Fluor 647, BioLegend #127610).
- **DNA/RNA sequences:** qPCR primer sequences are provided in the Methods (e.g., *Artn* primers). These sequences are not novel and were not deposited in a repository.
- **Cell materials:** Not applicable. No cell lines were used. Primary cells (e.g., BALF cells, lung immune cells, JNC neurons) were isolated from mice.
- **Experimental animals:** Yes. *Mus musculus* (mouse). Species, strains (including transgenic lines with JAX stock numbers/RRIDs), sex (male and female), and ages (typically ~6–16 weeks depending on experiment) are reported. Genotypes and breeding strategy (including littermate controls) are described.
- **Human participants:** Not applicable. No human participants were included.

**Design**

- **Study protocol (pre-registration):** Not applicable. The study was not pre-registered.
- **Laboratory protocol (public DOI/repository):** Not available. No protocols were deposited in an external protocol repository; procedures are described in the Methods.
- **Sample size:** Sample sizes are reported in figure legends and/or Methods. No a priori power analysis is reported.
- **Randomization:** Not applicable.
- **Blinding:** Not applicable.
- **Inclusion/exclusion criteria:** One RNA-seq sample (naïve group) was excluded as a PCA outlier; no other exclusions are reported. This is stated in the method section

**Ethics**

- **Animal research ethics:** Approved under institutional animal care protocols and conducted under CCAC guidelines. Approvals reported: McGill University (Mgcl-8184) and Queen’s University Animal Care Committee (UACC protocol 2384).
- **Human research ethics:** Not applicable.

**Analysis**

- **Attrition and exclusions:** No animal attrition is reported. One RNA-seq outlier sample was excluded (as above); otherwise, no exclusions.
- **Statistical methods:** Significance threshold reported (p ≤ 0.05). Statistical tests include Student’s t-test and one-way ANOVA in GraphPad Prism; RNA-seq differential expression was performed using DESeq2 (FDR-adjusted p-values, FDR < 0.05). Seurat-based analyses were conducted in R.

**Data availability**

- **RNA-seq data:** Raw and processed bulk RNA-seq data are deposited in GEO: **GSE298583**.
- **Other data:** Processed data are available in supplementary files; additional raw data are available via the provided Dropbox link and/or on request.
- **Behavioral/questionnaire data:** Not applicable.

**Code availability**

- **Code/scripts:** Not publicly available. Analyses used standard tools (GraphPad Prism; R/RStudio with DESeq2/Seurat), but scripts were not deposited.

**Reporting**

- **Standards/resources used:** In silico analyses reference established resources (e.g., ImmGen, Human Protein Atlas, CELLxGENE Discover) and enrichment analyses were performed using g:Profiler/GO-term.
- **Guidelines adherence statement:** Not applicable. No explicit statement of adherence to specific reporting guidelines (e.g., ARRIVE) is included in the Methods.

Key resources table – Experimental model organisms (Mus musculus)

| Reagent type (species) or resource | Designation | Source or reference | Identifiers | Additional information |
| --- | --- | --- | --- | --- |
| Strain, strain background (Mus musculus) | C57BL/6J | The Jackson Laboratory | Stock No: 000664; RRID: IMSR_JAX:000664 | Wild-type background strain used for experiments and/or breeding. |
| Strain, strain background (Mus musculus) | DTA^fl/fl^ (floxed diphtheria toxin A line; “DTAfl”) | The Jackson Laboratory | Stock No: 010527; RRID: IMSR_JAX:010527 | Cre-dependent DTA expression for genetic ablation when crossed to Cre driver lines. |
| Strain, strain background (Mus musculus) | DTA^fl/fl^ (floxed diphtheria toxin A line; “DTAfl”) | The Jackson Laboratory | Stock No: 009669; RRID: IMSR_JAX:009669 | Cre-dependent DTA expression for genetic ablation when crossed to Cre driver lines. |
| Strain, strain background (Mus musculus) | tdTomato^fl/fl^ (Ai14; “tdTomatofl”) | The Jackson Laboratory | Stock No: 007914; RRID: IMSR_JAX:007914 | Cre-dependent tdTomato reporter used to label/sort *Trpv1*⁺ neurons (*Trpv1*^cre/wt^::tdTomato^fl/wt^). |
| Strain, strain background (Mus musculus) | TRPV1^cre/cre^ | The Jackson Laboratory | Stock No: 017769; RRID: IMSR_JAX:017769 | *Trpv1* promoter-driven Cre; used to target *Trpv1*⁺ nociceptors for reporter labeling and/or DTA-mediated ablation. |
| Strain, strain background (Mus musculus) | Na_V_1.8^cre/cre^ | The Jackson Laboratory | Stock No: 036564; RRID: IMSR_JAX:036564 | Na_V_1.8 (*Scn10a*) promoter-driven Cre; used to target Na_V_1.8⁺ nociceptors for DTA-mediated ablation |

- **Key Resources – Antibodies (commercial reagents)**

| Reagent type (host species) | Designation (target [clone]) | Source (supplier) | Identifiers (catalog #; RRID) |
| --- | --- | --- | --- |
| Antibody (rat monoclonal) | Anti-mouse CD45 [clone *30-F11*] | BioLegend | Cat# 103128; RRID: AB_493715 |
| Antibody (rat monoclonal) | Anti-mouse CD90.2 (Thy1.2) [clone *53-2.1*] | BioLegend | Cat# 140307; RRID: AB_10643585 |
| Antibody (rat monoclonal) | Anti-mouse CD11b [clone *M1/70*] | BioLegend | Cat# 101243; RRID: AB_2561373 |
| Antibody (Armenian hamster mAb) | Anti-mouse CD11c [clone *N418*] | BioLegend | Cat# 117303; RRID: AB_313772 |
| Antibody (rat monoclonal) | Anti-mouse Ly6C [clone *HK1.4*] | BioLegend | Cat# 128004; RRID: AB_1236553 |
| Antibody (rat monoclonal) | Anti-mouse Ly6G [clone *1A8*] | BioLegend | Cat# 127610; RRID: AB_1134159 |
| Antibody (rat monoclonal) | Anti-mouse Siglec-F [clone *1RNM44N*] | Invitrogen (ThermoFisher) | Cat# 12-1702-82; RRID: AB_2637129 |
| Antibody (Armenian hamster mAb) | Anti-mouse TCR γ/δ [clone *GL3*] | BioLegend | Cat# 118128; RRID: AB_2562771 |
| Antibody (Armenian hamster mAb) | Anti-mouse TCR β [clone *H57-597*] | BioLegend | Cat# 127612; RRID: AB_2251161 |
| Antibody (rat monoclonal) | Anti-mouse CD19 [clone *1D3*] | BD Biosciences | Cat# 553783; RRID: AB_395047 |
| Antibody (mouse monoclonal) | Anti-mouse NK1.1 [clone *PK136*] | Invitrogen (ThermoFisher) | Cat# 25-5941-82; RRID: AB_469665 |
| Antibody (rat monoclonal) | Anti-mouse F4/80 [clone *BM8*] | Invitrogen (ThermoFisher) | Cat# 14-4801-82; RRID: AB_467558 |
| Antibody (rat monoclonal) | Anti-mouse FcεRIα [clone *MAR-1*] | BioLegend | Cat# 134318; RRID: AB_10640122 |
